# The effects of treatment in patients with childhood asthma on the elastic properties of the aorta

**DOI:** 10.5830/CVJA-2016-076

**Published:** 2017

**Authors:** Osman Bektaş, Zeki Yüksel Günaydin, Adil Bayramoğlu, Ahmet Kaya, Ahmet Karagöz, Recep Akgedik

**Affiliations:** Department of Cardiology, Faculty of Medicine, Ordu University, Ordu, Turkey; Department of Cardiology, Faculty of Medicine, Ordu University, Ordu, Turkey; Department of Cardiology, Faculty of Medicine, Ordu University, Ordu, Turkey; Department of Cardiology, Faculty of Medicine, Ordu University, Ordu, Turkey; Department of Cardiology, Faculty of Medicine, Giresun University, Giresun, Turkey; Department of Chest Diseases, Faculty of Medicine, Ordu University, Ordu, Turkey

**Keywords:** aortic stiffness, corticosteroids, bronchial asthma

## Abstract

**Introduction:**

The study aimed to investigate the effects of treatment in patients with childhood asthma on the elastic properties of the aorta and cardiovascular risk.

**Methods:**

The study was performed in 66 paediatric patients diagnosed with bronchial asthma (BA). All patients were administered the β_2_ agonist, salbutamol, for seven days, followed by one month of montelukast and six months of inhaled steroid treatment. All patients underwent conventionaltransthoracic echocardiographic imaging before and after treatment. Aortic elasticity parameters were considered to be the markers of aortic function.

**Results:**

Aortic elasticity parameters, including aortic strain (15.2 ± 4.8 and 18.8 ± 9.5%, p = 0.043), aortic distensibility (7.26 ± 4.71 and 9.53±3.50 cm^2^/dyn, p = 0.010) and aortic stiffness index (3.2 ± 0.6 and 2.8 ± 0.5, p = 0.045 showed significant post-treatment improvement when compared to pre-treatment values. Tricuspid annular plane systolic excursion (TAPSE) was also observed to improve after treatment (1.81 ± 0.38 and 1.98 ± 0.43, p = 0.049).

**Conclusion:**

The study demonstrated that when provided at appropriate doses, medications used in BA may result in an improvement in aortic stiffness.

## Introduction

Bronchial asthma (BA) is a chronic inflammatory disease of the airways. Exacerbations that develop as a result of bronchial hypersensitivity associated with chronic airway inflammation are accompanied by varying degrees of airway obstruction. The possible causes of airway obstruction include contraction of airway smooth muscles, hypersecretion of mucous, mucosal oedema, cell infiltration and epithelial desquamation.^1^ Studies have shown an association between systemic inflammation and the progression of asthma.^2^ It has also been established that pro-inflammatory cytokines such as tumour necrosis factor alpha (TNF-alpha), interleukin 6 (IL-6) and C-reactive protein (CRP) increase in patients with asthma.^2^,^3^ There is also evidence that supports the role of chronic inflammation in the aetiology of atherosclerosis.^4^ Chronic inflammation may even impair endothelial function and accelerate the progression of atherosclerosis.^5^ Endothelial dysfunction and arterial stiffness are two distinct components playing an important role in the pathophysiology of arterial diseases.

Nitric oxide (NO) released from the endothelial cells has been shown to contribute to arterial compliance and distensibility.^6^ Arterial stiffness consists of two components that are interrelated; the structural component is formed by the collagen elastin fibres and their associated molecules in the arterial medium, whereas the dynamic component is represented by the tonus of smooth muscle cells and depends on the vasoactive substances released by endothelial cells.^7^ In case of endothelial damage due to chronic inflammation or increased haemodynamic load, irrespective of the cause, it might be expected that arterial stiffness will also be affected. Indeed, arterial stiffness was shown to be elevated in a study performed on patients with BA.^8^ Aortic stiffness is a significant risk factor for cardiovascular mortality and morbidity.^9^

Due to their anti-inflammatory effects on the airways, β_2_-agonists, leukotriene receptor antagonists and inhaled corticosteroids (ICS) are used for the treatment of asthma. These medications carry the risk of enhancing the cardiovascular risk factors such as hypertension, hypercholesterolaemia, hypertriglyceridaemia and impaired glucose tolerance.^10^-^12^

Based on the fact that bronchial asthma is a chronic inflammatory disease, the present study aimed to demonstrate how aortic stiffness is affected by the treatment in paediatric patients with asthma, and to determine the possible impacts on cardiovascular risk.

## Methods

The study population consisted of children aged nine to 15, who were admitted to the paediatric ward and the chest diseases out-patient clinics of Ordu University between January and August 2012. A total of 240 patients who had been diagnosed with asthma without any treatment in the past three months or patients with newly diagnosed moderate-to-severe asthma were evaluated in the study.

In order to ensure standardisation, a treatment protocol of salbutamol 100 mcg 4 × 1 for 14 days, montelukast 5 mg/day for 30 days and budesonide 200 mcg inh 2 × 1 for six months was given to all patients according to the Global Initiative for Asthma (GINA) 2012.^13^ The study was continued with the patients in whom asthma was controlled with this treatment protocol.

One hundred and seventy-four patients were excluded because of uncontrolled BA, incompatibility with this protocol, severe asthma in whom intravenous/oral corticosteroid treatment or hospitalisation was required, need for salbutamol use in the control visit at the end of the second week, recurrence of the asthma attack, requirement for salbutamol treatment within six months, interruption of all triple treatment protocol at the end of fourth month, need for montelukast treatment for more than six months and lack of control echocardiography. Finally, 66 patients who had been given salbutamol 100 mcg inh 4 × 1 for 14 days, montelukast 5 mg tablets 1 × 1 for one month and budesonide 200 mcg inh 2 × 1 for six months were included in this study.

The right and left atria and ventricles were assessed using conventional transthoracic echocardiography and two-dimensional (2D) speckle tracking echocardiography. The initial echocardiographic evaluation was performed in the first six hours. The echocardiographic assessments were repeated by the same operators at the post-treatment sixth month. The preand post-treatment parameters were compared with each other.

All participants gave an informed consent and the study protocol was approved by the local ethics committee.

Chest X-ray, complete blood count, CRP and other biochemical parameters were evaluated in order to exclude conditions such as infection, bronchitis and bronchiectasis which can mimic asthma.

In accordance with the GINA 2012 guideline,^13^ bronchial asthma was diagnosed in the presence of 12% or more reversibility in forced expiratory volume 1 (FEV1) and 15% or more reversibility in peak expiratory flow (PEF) with the use of a salbutemol inhaler in the patients who had episodic breathlessness, coughing, chest congestion, wheezing and forceful expirium in their history. Only the patients with moderate and severe bronchial asthma were included. Mild bronchial asthma patients were excluded from the study.

Patients were assessed based on attack frequency over the previous three months for classification into controlled and uncontrolled asthma. Furthermore, their current level of asthma control was based on reduced lung function (PEFR), frequency of using reliever treatment, frequency of daytime symptoms, nocturnal symptoms or awakening because of asthma and any limitation of daily activities, including exercise.

Controlled asthma was defined as normal or near normal lung function results, no (≤ two times/week) need for reliever treatment, no (≤ two times/week) daytime symptoms, no nocturnal symptoms or awakening because of asthma, no limitation of daily activities including exercise and no exacerbation. Partially controlled asthma was also regarded as uncontrolled asthma. The patient was diagnosed as partially controlled asthma in the presence of any of the following criteria: reduced lung function < 80% of predicted or personal best, any limitation of activity, night-time cough, daytime symptoms more than twice per week and using rescue treatment more than twice per week. The condition was defined as uncontrolled asthma in the presence of three or more of the above criteria. Information on the patient’s age, gender, duration of bronchial asthma, and current treatment was obtained from each subject and checked against their case notes.

Conventional transthoracic echocardiographic imaging was performed by a double-blinded experienced operator while the patients were in the left lateral position and according to the recommendations of the American Echocardiography Society (AES).^14^ Left ventricular ejection fraction (LVEF, Simpson’s method), left ventricular systolic end-diameter, left ventricular diastolic end-diameter, left ventricle posterior wall thickness, interventricular septum thickness, left atrium diameters and volumes, and left ventricle volumes were evaluated. In addition to these echocardiographic assessments, M-mode, 2-D images, and colour-flow Doppler recordings of all patients were taken using a 2.5–3.5-MHz transducer of the echocardiography device (Philips IE33, Philips Medical Systems, Andover, MA).

After routine echocardiographic investigation, recordings of the ascending aorta were obtained from 3 cm above the aortic valve by the M-mode. Aortic diameters were calculated as the distance between the anterior and posterior wall inner edges of the aorta at systole and diastole. Systolic diameter of the aorta (AS) was recorded when the aortic wall was fully open. Diastolic diameter of the aorta (AD) was recorded simultaneously when the QRS peak was seen on electrocardiographic (ECG) recordings. Measurements were taken during five consecutive pulses and the mean was calculated. Echocardiographic assessments were repeated after three months of therapy and compared with the values recorded before therapy.

Aortic elasticity parameters were considered as markers of aortic function. Aortic systolic (AS) and aortic diastolic (AD) indices for each patient were calculated by dividing the systolic and diastolic aortic diameters by body mass index. Using these indices, elastic characteristics of the aorta were calculated as follows:

• Pulse pressure (mmHg) = SBP – DBP
• Aortic strain (%) = 100 (AS – AD)/AD
• Distensibility (cm^2^/dyn/10^3^) = 2 (AS – AD)/PP
• Aortic stifness index (ASI) = ln (SBP/DBP)/(AoS − AoD)/AoD

Where SBP = systolic blood pressure, DBP = diastolic blood pressure, PP = pulse pressure, AoS = aortic root end-systolic diameter, AoD = aortic root end-diastolic diameter.

## Statistical analysis

Continuous variables were expressed as mean ± SD or median (interquartile range). Categorical variables were expressed as percentages. An analysis of normality of the continuous variables was performed with the Kolmogorov–Smirnov test. To compare parametric continuous variables, paired samples t-test was used. To compare non-parametric continuous variables, the Mann–Whitney U-test or the Kruskal–Wallis test was used. Two-tailed p-values < 0.05 were considered as statistically significant. All statistical studies were carried out with the SPSS program version 20.0 for Windows.

## Results

The mean age of the patients was 11.6 ± 2.0 years (age range: 9–15 years) and 50% were males. Baseline characteristics are shown in Table 1. Echocardiographic and haemodynamic assessments did not show any difference between pre- and post-treatment values of heart rate (90.8 ± 7.7 and 89.5 ± 7.0 bpm, p = 0.320), systolic blood pressure (103.8 ± 10.6 and 102.6 ± 9.0 mmHg, p = 0.280), diastolic blood pressure (65 ± 7.2 and 66 ± 8.1 mmHg, p = 0.765), pulse pressure (37.5 ± 6.9 and 38.1 ± 6.3 mmHg, p = 0.442), and LVEF (68.5 ± 3.8 and 69.4 ± 3.4%, p = 0.427) (Table 2).

**Table 1:** Baseline characteristics of overall patients

*Baseline characteristics*	*n = 66*
Mean age (years)	11.6 ± 2.0
Male gender, n (%)	14 (56)
BMI (kg/m^2^)	17.1 ± 2.5
BSA (m^2^)	1.2 ± 0.5

BMI: body mass index, BSA: body surface area.

**Table 2:** Haemodynamic and echocardiographic findings before and after treatment of patients

*Variables*	*Before treatment*	*After treatment*	*p-value*
HR (bpm)	90.8 ± 7.7	89.5 ± 7.0	0.320
SBP (mmHg)	103.8 ± 10.6	102.6 ± 9.0	0.280
DBP (mmHg)	65 ± 7.2	66 ± 8.1	0.765
Pulse pressure (mmHg)	37.5 ± 6.9	38.1 ± 6.3	0.442
EF (%)	68.5 ± 3.8	69.4 ± 3.4	0.427
LVEDV (ml)	66.2 ± 13	67.9 ± 15	0.124
LVESV (ml)	24.3 ± 7.3	22.1 ± 6.7	0.278
LVMI (g/m^2^)	55.5 ± 19.4	56.2 ± 18.9	0.985
IVSD (cm)	6.9 ± 1.1	6.8 ± 1.8	0.848
PWD (cm)	6.8 ± 0.9	6.9 ± 1.0	0.789
Mitral E wave (m/s)	94.0 ± 14.5	95 ± 15.0	0.657
Mitral A wave (m/s)	64.2 ± 15.5	60.3 ± 19.1	0.433
Mitral E/A	1.56 ± 0.78	1.53 ± 0.95	0.678
DT	134.5 ± 30.7	144.7 ± 41.2	0.388
TAPSE	1.81 ± 0.38	1.98 ± 0.43	0.049
ePASP	19.2 ± 4.2	18.0 ± 5.3	0.230
LA diameter (mm)	2.4 ± 0.5	2.5 ± 0.6	0.568

HR: heart rate, bpm: beats per minute, SPB: systolic blood pressure, DBP: diastolic blood pressure, EF: ejection fraction, LVEDV: left ventricular end-diastolic volume, LVESV: left ventricular end-systolic volume, LVMI: left ventricular mass index, IVSD: interventricular septal defect, PWD: posterior wall thickness at end-diastole, DT: decelaration time, TAPSE: tricuspid annular plane systolic excursion, ePASP: estimated pulmonary artery systolic pressure, LA, left atrial.

Compared to the pre-treatment values, post-treatment TAPSE improved (1.81 ± 0.38 and 1.98 ± 0.43, p = 0.049) (Table 2). Aortic elasticity parameters including aortic strain (15.2 ± 4.8 and 18.8 ± 9.5%, p = 0.043), aortic distensibility (7.26 ± 4.71 and 9.53 ± 3.50 cm2/dyn, p = 0.010) and aortic stiffness index (3.2 ± 0.6 and 2.8 ± 0.5, p = 0.045) showed significant post-treatment improvement when compared to pre-treatment values (Table 3).

**Table 3:** Aortic elastic properties (strain, stiffness index, distensibility) of patients before and after treatment

*Properties*	*Before treatment*	*After treatment*	*p-value*
Aortic velocity (m/s)	0.8 ± 0.1	0.9 ± 0.2	0.563
Aortic diameter in systole (mm)	20.9 ± 2.9	21.2 ± 2.5	0.203
Aortic diameter in diastole (mm)	18.5 ± 2.2	18.6 ± 2.4	0.812
Aortic strain (%)	15.2 ± 4.8	18.8 ± 9.5	0.043
Aortic distensibility (cm^2/^dyn)	7.26 ± 4.71	9.53 ± 3.50	0.010
Aortic stiffness index	3.2 ± 0.6	2.8 ± 0.5	0.045
Cardiac output (l/min)	3.85 ± 1.2	4.08 ± 1.1	0.032
Stroke volume (ml)	42 ± 5.0	45 ± 4.2	0.044

## Discussion

The findings of this study demonstrate that aortic stiffness improved after appropriate dosage medications in asthma patients. Such improvement may be associated with the antiinflammatory effects of therapy. Additionally, the medication had positive effects on right ventricular systolic functions and cardiac output. We suggest that the decrease in aortic stiffness might have contributed to this improvement, and we might have treated the negative cardiac effects while treating bronchial asthma.

BA is the most common reason for paediatric respiratory disorders and it is a significant cause of mortality and morbidity.^1^,^13^ Exposure to BA-associated repetitive hypoxia results in sustained pulmonary vasoconstriction and long-term obstruction of the pulmonary veins. In addition, pulmonary hypertension develops, resulting in right ventricular hypertrophy and enlargement, which is also known as cor pulmonale.^15^ In our study, the significant post-treatment increase in TAPSE can be interpreted as improvement in right ventricular function.

The negative effects of BA on right heart functions are well known; however, on the contrary to this well-known pathology, there is relatively limited knowledge about how left heart chambers are affected by this condition or how they change after therapy. In a study assessing left ventricular function during acute asthma exacerbations, it has been demonstrated that transmitral peak A wave increased and E/A ratio decreased during an acute severe asthma exacerbation, which implies the development of left ventricular diastolic dysfunction.^16^ In summary, it can be said that both the right and the left heart functions are negatively affected during the course of BA.

In our study, in addition to TAPSE, cardiac output and stroke volume were significantly increased after treatment but there was no significant difference in E/A ratio. These findings can be interpreted as that treatment improves both right and left heart functions. However similar E/A ratios after BA treatment indicate that the medication had no effect on left ventricle diastolic function. A future study designed using novel diastolic parameters and control group could provide clearer results.

Apart from the direct impact of hypoxia on impairment of the cardiac function and aortic stiffness in BA, some other aetiological factors may also contribute to this situation. Indeed, Massoud et al. found evidence showing the effects of chronic and sustained inflammation on myocardial function in patients with severe asthma. Inflammatory mediators also increase with inflammation when the respiratory symptoms appear, and it is known that some of these mediators have the potential to significantly depress cardiac contractility (TNF-alpha, IL-1β, IL-2, IL-6, IL-8, IL-10).^17^ The patients’ symptoms recover and the frequency of repetitions decrease with medications especially steroids, and this might contribute to the decrease in mediator secretion, which in turn may have resulted in an improvement of cardiac functions and aortic stiffness at the same time.

In our study, the primary target was to evaluate the relationship between medication and aortic stiffness and no patient had any other chronic inflammatory disease such as diabetes, hypertension or congestive heart failure that may have affected aortic stiffness.

In acute severe asthma, cardiovascular function significantly changes as a result of the direct effects of BA or secondary to the drug therapy (β_2_-adrenergic receptor agonists and steroids in particular).^18^ Several studies have demonstrated that there is a relationship between increased mortality rates and β_2_-agonists in patients with BA. Moreover, there have been cases of sudden cardiac death^19^,^20^ and congestive heart failure^21^ related to β_2_-agonists.

However, in the present study, we administered the β_2_-agonist, salbutamol, for only seven days to ease the symptoms, and thereafter we excluded the patients who required additional β_2_-agonist therapy. We administered only one month of montelukast. After the first month, patients received only ICS therapy for five months. Therefore our study is also important in terms of assessing the effects of inhaled corticosteroids (ICS) on aortic stiffness. The cardiac risk that develops after corticosteroid administration is related to the dosage. ICSs are commonly used in BA. Although ICSs are considered to have far less systemic absorption and possible systemic side effects, there are still some concerns. Therefore, they are recommended to be used in the lowest possible therapeutic doses.^22^

Lorenzo et al. noted that the risk of acute myocardial infarction (MI) increases in patients using oral corticosteroids; however, there is no such risk increase in patients using ICS.^23^ Conversely, acute MI risk in patients with BA decreased with the use of ICS therapy in another study.^10^ In general, this finding supports the studies demonstrating the role of inflammation in the aetiology of atherosclerosis, as well as the results of our study.^7^ This might be explained by the fact that the dose that enters into the systemic circulation upon ICS therapy is low enough to be below the level to exacerbate cardiovascular risk, but still at a level to have the potential to repress the inflammation associated with atherosclerosis.

In line with these studies, an animal study demonstrated that the circulating levels of cholesterol and triglycerides did not increase upon administration of corticosteroids, whereas plaque formation and progression decreased.^24^ In another study, a decreased cardiovascular mortality rate was demonstrated upon ICS administration in patients with asthma.^25^ Corticosteroids show cardiovascular effects through direct inhibition of expression of the vascular adhesion molecules, in addition to non-transcriptional activation of endothelial nitric oxide synthase.^26^ As is already known, nitric oxide (NO) released from the endothelium contributes to arterial compliance and distensibility.^6^ Chronic inflammation and oxidative stress are known to co-exist in BA.^27^ The levels of reactive oxygen species such as hydroxyl radicals, superoxides, and peroxides are elevated in BA patients who have inflammation.^28^ Chronic inflammation is associated with endothelial dysfunction, atherosclerosis and arterial stiffness, all of which are risk factors for future cardiovascular events.^29^

In paediatric BA patients who do not use ICS therapy, carotid intima–media thickness (CIMT) was increased compared to the control group and a positive correlation was noted between CIMT and total oxidant status.^28^ In another study, BA patients treated with ICSs had decreased carotid atherosclerosis compared to the control group.^30^ The results of the present study are not surprising, considering previous studies and keeping in mind that atherosclerosis starts during childhood. However, considering the age group examined in this study, it does not seem right to attribute the improvement in aortic stiffness after ICS therapy only to the role of inflammation in the aetiopathogenesis of atherosclerosis. The suppression of the negative cardiac effects of inflammatory steroids by ICS therapy may be the dominant mechanism leading to healing, since studies have demonstrated that steroids improve aortic stiffness through a similar mechanism.^31^

Some studies have established that assessment of aortic stiffness can be used for early detection of atherosclerosis. Moreover, aortic stiffness has been shown to increase with advanced age, and in the presence of various conditions such as hypertension, atherosclerosis, β-thalassaemia, smoking, obesity, Marfan syndrome and Kawasaki disease.^32^-^34^

Finally, in the present study the significant improvement in TAPSE and haemodynamic recovery can be explained by the anti-inflammatory effects of medications especially steroids, in addition to being a direct outcome of the decrease in the frequency of acute BA exacerbations; in other words, a decrease in the duration of exposure to hypoxia. Hence, it can be speculated that in addition to improving aortic stiffness, BA medication may also improve right ventricular systolic function. Previous studies have demonstrated that the presence of BA in paediatric populations can result in subclinical ventricular dysfunction as detected by tissue Doppler imaging.^35^

The limitations of this study include the small number of patients, the treatment regimen including both β_2_ mimetics, montelukast and steroids, the short duration of treatment and absence of a control group. The number of patients was 60 at the beginning of study; however, 15 patients who required re-initiation of β_2_ mimetics apart from the standard therapy were excluded. Patients were administered β_2_ mimetics and montelukast only at the beginning of therapy, and thereafter they were monitored on steroids alone. Therefore, therapies other than steroids can be considered not to have affected aortic stiffness during this period. A control group was not deemed necessary, since the purpose of the study was to assess the effects of ICS therapy on aortic stiffness in BA patients.

## Conclusion

Contrary to the usual concerns about cardiac effects, medications to treat asthma can be used safely in childhood BA and may improve aortic stiffness through their anti-inflammatory effects, and additionally by decreasing exposure to hypoxia. Moreover, early diagnosis of BA and the initiation of therapy may contribute to a decrease in cardiac mortality and morbidity rates; however, additional long-term studies are required to develop accurate conclusion on this subject.
